# Detection of *E. coli* Bacteria in Milk by an Acoustic Wave Aptasensor with an Anti-Fouling Coating

**DOI:** 10.3390/s22051853

**Published:** 2022-02-26

**Authors:** Sandro Spagnolo, Brian De La Franier, Katharina Davoudian, Tibor Hianik, Michael Thompson

**Affiliations:** 1 Faculty of Mathematics, Physics and Information, Comenius University, Mlynská dolina F1, 842 48 Bratislava, Slovakia; sandrospagnolo1@gmail.com (S.S.); tibor.hianik@fmph.uniba.sk (T.H.); 2 Department of Chemistry, University of Toronto, 80 St. George Street, Toronto, ON M5S 3H6, Canada; brian.delafranier@mail.utoronto.ca (B.D.L.F.); k.davoudian@mail.utoronto.ca (K.D.)

**Keywords:** *E. coli*, biosensor, anti-fouling, MEG-Cl, aptamer, acoustic wave sensor, milk

## Abstract

Milk is a significant foodstuff around the world, being produced and consumed in large quantities. The safe consumption of milk requires that the liquid has an acceptably low level of microbial contamination and has not been subjected to spoiling. Bacterial safety limits in milk vary by country but are typically in the thousands per mL of sample. To rapidly determine if samples contain an unsafe level of bacteria, an aptamer-based sensor specific to *Escherichia coli* bacteria was developed. The sensor is based on an ultra-high frequency electromagnetic piezoelectric acoustic sensor device (EMPAS), with the aptamer being covalently bound to the sensor surface by the anti-fouling linker, MEG-Cl. The sensor is capable of the selective measurement of *E. coli* in PBS and in cow’s milk samples down to limits of detection of 35 and 8 CFU/mL, respectively, which is well below the safe limits for commercial milk products. This sensing system shows great promise for the milk industry for the purpose of rapid verification of product safety.

## 1. Introduction

Milk is a highly important foodstuff, with the European Union producing over 160 million tons of milk each year [1]. For milk to be safe for consumption it must be reasonably free of harmful microbes, such as *Escherichia coli* (*E. coli*) or *Staphylococcus aureus* (*S. aureus*), which could render illness in consumers. For example, foodborne Shiga toxin-producing *E. coli* (STEC) can cause mild to severe clinical conditions, including acute gastroenteritis and hemolytic uremic syndrome [2,3]. In Europe, approximately 6% of STEC cases result from dairy consumption [2]. An increasing interest in raw drinking milk (RDM) poses further health risks from STEC and other foodborne pathogens. Pathogenic contamination in RDM can have additional health risks, especially as bovine antibiotic use can lead to antimicrobial-resistant bacteria in milk [4,5].

In the European Union countries, the safe limit of bacterial contamination in milk is 10^5^ colony-forming units/mL (CFU/mL), with less than 1.5 × 10^4^ CFU/mL being desirable [6]. The number of bacteria in milk samples is generally measured by using agar plate counts [7], with typically thousands of bacterial colonies found per milliliter of sample. This is achieved by spreading a small amount of sample on an agar plate, either on its own or diluted, and incubating it at 37 °C overnight. Any live bacteria should grow into a visible colony in that time and can be counted either by hand or using computer imaging software. However, this requirement of an overnight assay to obtain results followed by accurate counting of small colonies makes this measurement slow and somewhat unreliable.

The development of a rapid biosensor, which could quantify the number of bacteria present in milk samples, would greatly improve the accuracy and rapidity of milk safety evaluation to determine if samples contain an unsafe quantity of bacteria. To do so, a sensor probe for the bacterial species of interest would need to be covalently bound to a sensor surface.

Among the perspective probes for recognition of bacteria, the DNA aptamers are of substantial interest. They are single-stranded oligonucleotides developed by combinatorial chemistry using the method called SELEX (Systematic Evolution of Ligands by Exponential Enrichment). This method has been originally developed for the detection of proteins, such as thrombin or small molecules, for example, fluorescence dyes. However, it can also be useful for the detection of whole bacteria, cells, or viruses. This modification of SELEX is known as Cell-SELEX. In this method, the aptamers are selected in particular for a certain protein in the cell membrane [8]. The aptamer in a solution folds into a 3D structure, forming a binding site for the corresponding analyte. In contrast with antibodies that were also used for the development of affinity bacterial sensors [9], the aptamers have several advantages. They can be developed in vitro for a practically unlimited number of different compounds. They are also not immunogenic and are more stable than antibodies. Once the aptamer oligonucleotide sequence is developed it can be reproduced with high precision using standard oligonucleotide synthesis. Aptamers can be modified by various compounds which increase their stability toward cleavage by nucleases and allow their immobilization at various surfaces [10].

The aptamers sensitive to *E. coli,* which is a very common milk contaminant [11,12,13,14], have been used so far mostly for the development of electrochemical [10,15] and optical [16,17] aptasensors. There is, however, only a limited number of papers published so far on *E. coli* detection by acoustics methods. In the paper by Urmann et al. [18] the thiolated aptamers with the same sequence as those used in our work have been chemisorbed on a quartz crystal microbalance (QCM) transducer. The limit of detection (LOD) for *E. coli* has been determined as 10^6^ CFU/mL. Yu et al. [13] reported a LOD of 1.46 × 10^3^ CFU/ mL for *E. coli* O157:H7 detection by QCM. Most recently, Khobragade et al. [19] achieved a LOD of 10^4^ CFU/mL for *E. coli* using aptasensors based on a non-linear quartz crystal resonator method (QCR).

In this work, we applied DNA aptamers specific to *E. coli* for the development of an acoustic wave sensor based on ultrathin quartz discs called an electromagnetic piezoelectric acoustic sensor (EMPAS) [20], which operates at very high frequencies, around 1 GHz. To obtain accurate measurements, any non-specific adsorption of foreign material, often termed fouling, needs to be prevented so a signal change is due to the analyte of interest [21]. Many strategies have been employed to reduce sensor fouling, with the use of surface-assembled monolayers being very common [22,23,24].

We have developed a very promising ultra-thin anti-fouling coating, from hydrolyzed 2-(3-trichlorosilylpropyloxy)-ethyl trifluoroacetate (MEG-OH), which has successfully been used to dramatically reduce fouling from various biological agents [25,26,27]. To attach sensor probes to surfaces, a similar compound was developed which contains a highly reactive acyl chloride terminal functionality, 3-(3-(trichlorosilylpropyloxy) propanoyl chloride (MEG-Cl) [28]. This coating was found to reduce surface fouling of EMPAS sensors from milk to a large degree, opening the possibility of developing sensors that can function in real samples [28].

In this work, MEG-Cl was used to bind the *E. coli* selective aptamer to the sensing surface of EMPAS quartz crystals to quantify the number of bacteria in either buffer or milk samples. The goal of research on this sensor is to not only detect the presence of *E. coli* bacteria in milk samples, but also to quantify the number of bacteria on a selective basis.

## 2. Materials and Methods

### 2.1. Materials

MEG-Cl was synthesized according to previously published methods [28,29]. Anhydrous toluene and pyridine were purchased from Sigma–Aldrich (St. Louis, MO, USA). The DNA aptamer was obtained in lyophilized form from Biosearch Technologies (Risskov, Denmark) and is represented by an amine-terminated oligonucleotide at the 5′ end. The sequence of the aptamer used has been taken from [30]: 5′-NH_2_-ATC CGT CAC ACC TGC TCT ACG GCG CTC CCC AAC AGG CCT CTC CTT ACG GCA TAT TAT GGT GTT GGC TCC CGT AT-3′. Ethanol was obtained from Caledon Laboratory Chemicals (Georgetown, ON, Canada). All chemicals were used without further purification. UHT cow’s milk, 3.5% fat, was purchased from Walmart (Toronto, Canada). Adhesion experiments were carried out with *Escherichia coli* DH5α, *Pseudomonas aeruginosa* PAO1, and *Staphylococcus aureus* KR3 purchased from the University of Toronto Medstore (Toronto, ON, Canada).

### 2.2. Preparation of Aptamer Stock Solution

The DNA aptamer was originally selected for the outer membrane proteins of the *E. coli* 8379 strain [30]. However, because these proteins are common for various *E. coli* strains [30], including those used in our experiments (*E. coli* DH5α), we applied this aptamer in the sensor development. Before use, the aptamer was re-suspended in a pH 8.0 TE buffer containing 10 mM Tris-Cl and 1 mM EDTA in DNase-free water to obtain a 100 μM concentration of the initial stock solution. Subsequently, a portion of the stock solution thus obtained was diluted 10 times to a working concentration of 10 μM with PBS buffer and divided in 100 μL volume aliquots into single-use micro-vials, stored at −20 °C until use at the desired concentration for only one experiment.

### 2.3. Cleaning and Surface Modification of Quartz Crystals

The procedures in Section 2.3 have been previously published in a paper for which this work is a continuation [29]. Quartz crystals (AT-cut, 13 mm in diameter, t = 83 μm thickness, 20 MHz fundamental frequency) purchased from Laptech Precision Inc. (Bowmanville, Ontario, Canada), were rinsed according to the previously published methods in 1% sodium dodecyl sulphate (SDS, Sigma-Aldrich, St. Louis, MO, USA), then rinsed by tap water, and subsequently in distilled water. The crystals were then individually soaked in 6 mL pre-heated to 90 °C Piranha solution (3:1 *v*/*v* mixture of 98% H_2_SO_4_ and 30% H_2_O_2_), using a water bath for 30 min. They were then rinsed in distilled water followed by methanol and sonicated for 2 min in another portion of methanol. After this, they were rinsed again with methanol, then dried by placing them in an oven at 150 °C for 2 h. They were then plasma cleaned for 15 min to increase surface hydroxylation and transferred into a humidity chamber (70–80% RH, room temperature) for overnight surface hydration.

The following day the crystals were submerged in toluene containing MEG-Cl (1000:1) in an inert (N_2_) and anhydrous (P_2_O_5_) atmosphere in glass vials. The sealed vials were then rotated for 90 min after which the crystals were rinsed with toluene. They were then sonicated in toluene for 5 min, rinsed with deionized water, and dried under a stream of nitrogen, leaving MEG-Cl crystals.

**Scheme 1:**
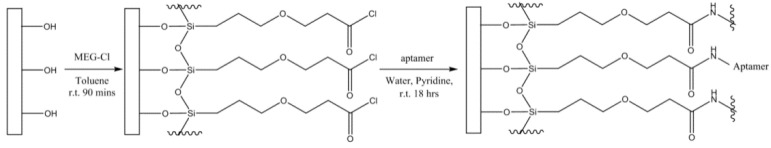
Surface modification with MEG-Cl followed by extension with N_α_,N_α_-bis(carboxymethyl)-L-lysine (ab-NTA) to form a MEG-Aptamer surface coating.

Some MEG-Cl crystals were extended with the aptamer by creating a dilute aliquot of 1.3 μM aptamer solution in deionized water (750 μL volume). The water solution was added to each test tube along with pyridine (0.25 mL) to have a ratio of 3:1 water solution: pyridine, and a 1 μM final aptamer concentration. The test tubes were then placed on a spinning plate overnight. These crystals, now modified with MEG-Aptamer, were rinsed with deionized water and dried under a gentle N_2_ flow. The main steps in the preparation of the antifouling sensing layer at the surface of the EMPAS crystal are summarized in Scheme 1.

### 2.4. EMPAS Measurements

EMPAS measurements were performed in either 10 mM, pH 7.4 phosphate-buffered saline (PBS, 10 mM Na_2_HPO_4_, 154 mM NaCl), or UHT cow’s milk. Bacteria were suspended in solution by first growing up a pre-culture of the chosen bacteria at 37 °C overnight in Lysogeny Broth. Then, 1 mL of pre-culture was then centrifuged at 14,500 RPM for 5 min to pellet the bacteria. The bacteria pellet was then resuspended in 1 mL of PBS, after which the solution’s optical density at 600 nm was measured by the UV-Vis spectrometer UV-1600PC (VWR International, Mississauga, Canada). The CFU was calculated from the optical density according to the following papers [31,32,33]. For samples in PBS, the resuspension was diluted to the desired concentration. For samples in milk, the resuspension was once again pelleted, the PBS removed, and the bacteria resuspended again in milk at the desired concentration.

Experimental performance of the EMPAS has been described previously [34]. After the standard set-up of the EMPAS, a bare, MEG-Cl coated, or MEG-Aptamer SAM-coated quartz crystal was installed in a custom flow-through cell, and PBS buffer was flowed over the crystal at a rate of 50 μL/min.

An ultra-high frequency of 984 MHz was used to perform the EMPAS measurements. The frequency was allowed to stabilize under PBS flow, after which 250 μL of either PBS or milk sample containing a known concentration of bacteria was injected into the cell system by transferring the inlet tube to a vial containing the sample, before switching back to PBS. PBS buffer flow was continued to rinse the crystal surface of any loosely bound material. Once the frequency signal stabilized again, the experiment was stopped, and the frequency shift was calculated. The experiment was repeated in triplicate for each type of crystal.

## 3. Results

### 3.1. Sensing of E. coli in PBS and Milk

**Figure 1:**
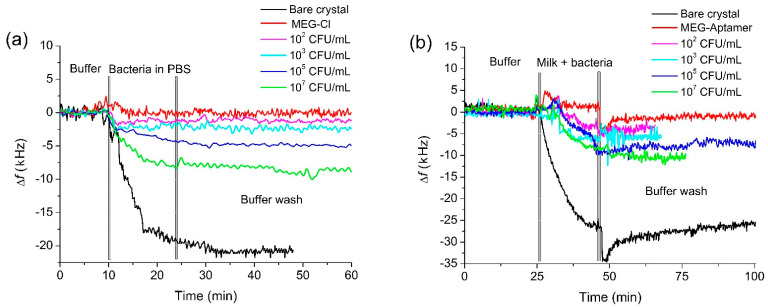
Plots showing the change in frequency, Δf, vs. time for interaction of *E. coli* at various concentrations in PBS buffer (**a**) or in UHT cow’s milk (**b**) to either a bare crystal, a crystal covered by MEG-Cl linker, as well as MEG-Aptamer-coated crystals. Bare and MEG-Cl coated crystals were exposed to 10^8^ CFU *E. coli*, while MEG-Aptamer coated crystals were exposed to the concentrations specified in the legends.

In the first series of experiments, we studied the kinetics of the changes in the frequency of the bare piezocrystal as well as those modified by antifouling MEG-Cl linker with attached DNA aptamers following interaction with various concentrations of *E. coli* in a PBS (Figure 1a) as well as in UHT cow’s milk (Figure 1b). As can be seen from Figure 1a, the addition to the bare crystal of a relatively large concentration of *E. coli* (10^8^ CFU/mL) in a buffer resulted in a sharp decrease of the resonant frequency (17 ± 4 kHz), which is evidence of non-specific adsorption of bacteria to the surface without coverage of antifouling layer. This high concentration was chosen to saturate the surface and show the maximum amount of potential fouling, and the change that would result from it.

However, a much smaller decrease in the frequency (0.5 ± 0.8 kHz) has been observed when the same *E. coli* concentration has been flowed over the surface of the piezocrystal modified by MEG-Cl linker. It is also clearly seen that with increased concentration of *E. coli* in a buffer added to the crystal surface covered by MEG-Aptamers, a decrease of the frequency was more significant. Similar results were obtained when instead of PBS the UHT milk had been used (Figure 1b). As can be seen from Figure 1b, the flow of the milk on the surface of bare crystal resulted in a sharp decrease in frequency due to non-specific adsorption of the milk components. However, the crystal covered by aptamers linked to MEG-Cl protects the piezoelectric transducer from substantial non-specific interactions. Although a certain decrease of resonant frequency was observed for milk flowed on the MEG-Aptamer surface, which is evidence of the matrix effect of milk components. The addition of the milk spiked by various concentrations of *E. coli* resulted in a decrease of the frequency when exposed to MEG-Aptamer crystals.

**Figure 2:**
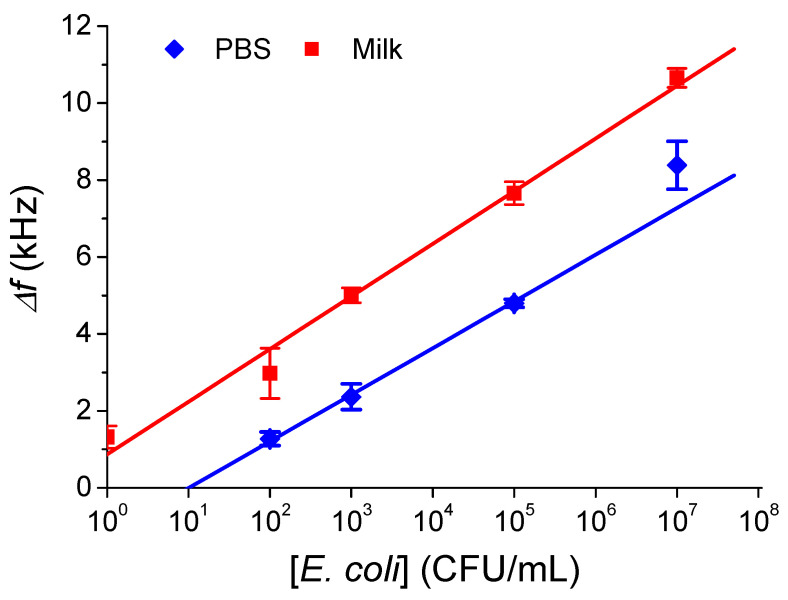
Frequency change, Δ*f*, of EMPAS instrument for MEG-Aptamer-coated crystals after exposure to varying concentrations of *E. coli* in either PBS (blue diamonds) or milk (red squares) in log scale. Lines represent linear fits performed by OriginPro, version 7.5 (OriginLab Corporation, Northampton, MA, USA) with the following parameters: Δ*f* (kHz) = 1.21 log [*E. coli*] *−* 1.22, R^2^ = 0.995 (PBS) and Δ*f* (kHz) = 1.37 log [*E. coli*] *+* 0.87, R^2^ = 0.994 (Milk).

The results are summarized in Table 1 and in Figure 2. The sensors’ data were normalized to the average frequency of the crystal immediately prior to sample exposure, and the change in frequency after exposure was measured as the average of the final 5 min of readings after a suitable amount of wash-off in PBS. Such exposure always resulted in a decreased signal, however, for displaying the data, the frequency change, Δ*f*, is stated in positive terms (Table 1, Figure 2).

When MEG-Aptamer-coated crystals were exposed to various concentrations of *E. coli* in either PBS or milk, a logarithmic trend was observed with quantification being possible at all measured concentrations (Figure 2).

For milk samples, there was a 1.3 kHz change due to the milk, which was present for all concentrations of bacteria tested. Using the standard deviation of noise (signal-to-noise ratio: S/N = 3) for a representative EMPAS experiment of approximately 126 Hz, a theoretical LOD for *E. coli* in PBS of 35 CFU/mL was calculated as well as a limit of quantification (LOQ) of 146 CFU/mL. These values are even lower in milk at 8 and 34 CFU/mL for the LOD and LOQ, respectively.

The obtained values are much lower in comparison with so far reported acoustic wave biosensors for *E. coli* detection, as discussed in the Introduction. As it is evident from the comparison of the calibration curve for *E. coli* detection in a PBS and in UHT cow’s milk, there is a certain matrix effect of milk components that probably interact with the MEG-Aptamer coating. On the other hand, the configuration of the aptamer and consequently the binding properties can change in different environments. This effect requires additional analysis, for example, determination of the constant of association, *K_d_*. For this purpose, we analyzed the binding properties of bacteria to the aptamers by means of fitting the binding curves constructed in a linear concentration scale by Langmuir isotherm [35]:

Δ*f* = (Δ*f*)_max_ [*c*/(*K_d_ + c*)] (1)

where (Δ*f*)_max_ is the maximal frequency changes, *K_d_* is the constant of dissociation and *c* is the concentration of bacteria. The lower the value of *K_d_* is, the stronger the complex aptamer-bacteria is. It is convenient to present Equation (1) in the Lineweaver–Burk coordinates (1/Δ*f* vs. 1/*c*):

1/Δ*f* = *K_d_*/(Δ*f*)_max_·*c* + 1/(Δ*f*)_max_ (2)

**Figure 3:**
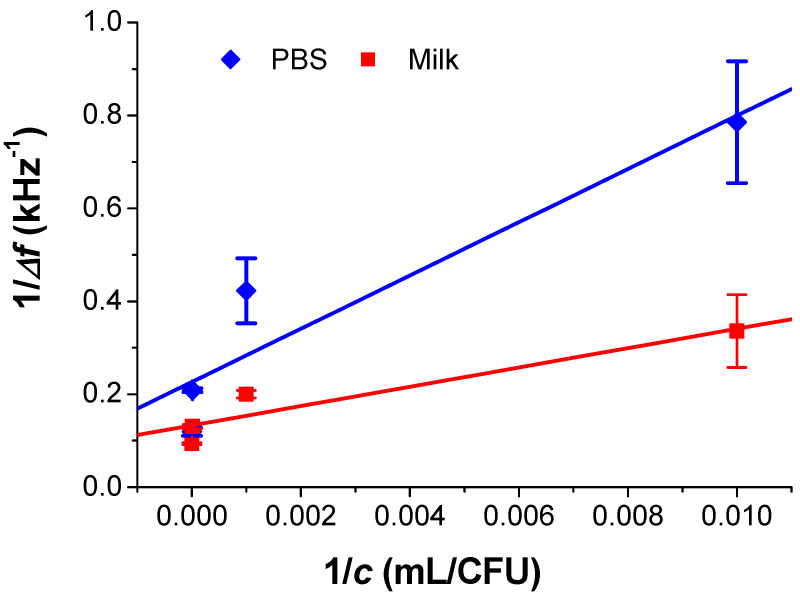
The Lineweaver–Burk plot of the frequency changes (1/Δ*f*) vs. reverse concentration of *E. coli* (1/*c*) for aptasensor-based on DNA aptamers with antifouling properties. Lines represent linear fits performed by OriginPro, version 7.5 (OriginLab Corporation, Northampton, MA, USA) with the following parameters: 1/Δ*f* (kHz^−1^) = (57.33 ± 14.88)/*c* (CFU^−1^·mL·kHz^−1^) + (0.23 ± 0.07) (kHz^−1^), R^2^ = 0.939 (PBS) and 1/Δ*f* (kHz^−1^) = (20.78 ± 5.10)/*c* (CFU^−1^·mL·kHz^−1^) + (0.13 ± 0.03) (kHz^−1^), R^2^ = 0.945 (Milk).

This plot is shown on Figure 3. By fitting the calibration curves in Figure 3 we obtained the following *K_d_* and (Δ*f*)_max_ values for binding the bacteria to the aptamer in a buffer (*K_d_* = 253.6 ± 65.8 CFU/mL; (Δ*f*)_max_ = 4.4 ± 1.5 kHz) and in milk (*K_d_* = 159.8 ± 39.2 CFU/mL; (Δ*f*)_max_ = 7.5 ± 1.4 kHz), respectively. It can be seen that the *K_d_* in milk is lower in comparison with those in PBS. We can speculate that one of the possible reasons (in addition to the matrix effect) can be certain changes in the conformation of the aptamers, which may improve the binding affinity. This is, however, only a preliminary assumption. More detailed analysis is therefore required, for example using circular dichroism or molecular dynamic simulations methods.

### 3.2. Testing the Selectivity of the Aptasensor

**Figure 4:**
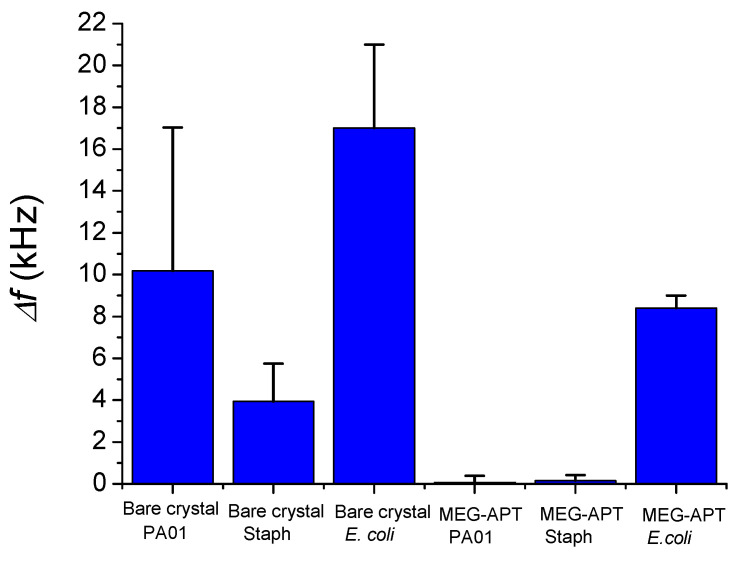
Change in frequency, Δ*f*, for unmodified and MEG-Aptamer (MEG-APT) modified EMPAS crystals after exposure to either *P. aeruginosa* (PAO1), *S. aureus* (Staph) or *E. coli* in PBS. Concentrations of bacteria: 10^8^ CFU/mL (bare crystal), 10^8^ CFU/mL (PAO1 and Staph), 10^7^ CFU/mL (*E. coli*) (MEG-Aptamer).

To determine if the developed aptasensor was selective for *E. coli* bacteria, two additional species *P. aeruginosa* (PAO1) and *S. aureus* (Staph) were exposed in high concentrations (1 *×* 10^8^ CFU/mL) to unmodified EMPAS crystals and MEG-Aptamer crystals in a PBS (Figure 4). We used high concentrations of the bacteria, considering that at lower concentrations the interaction of these bacteria with MEG-Aptamer layers was negligibly small. As with previous measurements, the change in frequency is reported in positive values for visual clarity. From these measurements, PAO1 and Staph are both able to foul unmodified EMPAS crystals, though to a lesser extent than *E. coli*. PAO1 causes a larger change in frequency compared to Staph, suggesting a greater amount of fouling, which is to be expected based on a previous study [27]. In addition, the variance in fouling is quite high, which is expected since there is no specific attachment during these experiments.

Meanwhile, MEG-Aptamer-coated crystals show virtually no signal change after exposure to either PAO1 or Staph, with their standard deviations overlapping zero Hz in change.

## 4. Discussion

The primary purpose of the developed aptasensor is the detection of *E. coli* in solution. To determine if this was possible, EMPAS crystals were coated with MEG-Cl, which was further extended with the *E. coli* selective aptamer. The surface fouling observed on unmodified crystals from *E. coli* and from milk would make accurately quantifying the concentration of bacteria present extremely challenging as the fouling would cause large and unexpected changes in the signal.

As the MEG-Cl and MEG-Aptamer-coated crystals are capable of dramatic reductions in fouling, any signal change in sensor measurements can be attributed to the analyte of interest binding to the probe, and not as a result of fouling. This is very important in any biosensor measurement and suggests that MEG-Cl is a viable anti-fouling linker for these systems.

The logarithmic trend that results from increasing concentrations of *E. coli* in both PBS and milk is nearly identical when the 1.3 kHz fouling from milk is accounted for. As such, quantification of *E. coli* in both media is possible. It is hypothesized that the logarithmic fit is a result of the surface packing of the large *E. coli* cells. As more and more cells are bound to the surface, this would greatly reduce the binding ability of further cells due to being blocked by previous cells. Further experiments, such as fluorescently staining the crystals after measurement and imaging them under microscopy, would need to be performed to test this hypothesis.

The EMPAS system itself would be difficult to implement in milk production facilities. A different biosensing device based on MEG-Cl and an *E. coli* aptamer could likely be created which would function to rapidly quantify bacterial contamination in milk samples.

When it comes to exposure of EMPAS crystals with other species of bacteria, the MEG-Aptamer-coated crystals show virtually no signal change after exposure to either PAO1 or Staph. The lack of signal change shows that the aptamer is unable to bind to these bacteria species and that the MEG-Cl coating prevents the bacteria from surface-fouling. This result indicates that the developed aptasensor is specific to *E. coli* and is likely able to function in the presence of other bacterial species, though confirmatory competition studies have yet to be performed.

As previously mentioned in the Introduction, existing acoustic wave-based aptasensors for *E. coli* achieved a LOD for *E. coli* several orders of magnitude higher than what was observed for the EMPAS-based aptasensor in this work. Moreover, in these papers, the aptasensors were not validated in milk samples. Thus, the EMPAS aptasensor revealed superior sensitivity for *E. coli* and allows the detection of bacteria in raw and undiluted milk samples. We should also emphasize that the sensor reported in this work has better or comparable sensitivity also in comparison with so far published electrochemical and optical sensors for detection of *E. coli* as it is evident from Table 2. However, no one sensor has been validated in not diluted milk samples.

The present work strongly suggests that a biosensor using MEG-Cl as an anti-fouling surface linker in combination with an aptamer selective to the bacteria of choice, in this case, *E. coli*, could be developed and used to rapidly quantify bacteria in real-world samples. Such a detection system could greatly increase the safety of milk worldwide, as unsafe levels of bacteria could be rapidly determined, and contaminated milk samples removed from sale.

## 5. Conclusions

The developed aptamer-based sensor using MEG-Cl as an anti-fouling surface linker was able to successfully detect and quantify the presence of *E. coli* bacteria in PBS and milk samples. The sensor was found to drastically reduce fouling of bacteria and milk to the crystal surface, allowing for the selective measurement of *E. coli* in the samples. The limit of quantification of 34 CFU/mL in milk samples is well below the safe limit for bacteria allowed in milk products in the EU, suggesting that such a sensor would be applicable in the milk industry. In addition, the near-complete lack of signal observed for the sensor when exposed to competing bacteria species *P. aeruginosa* and *S. aureus* shows that the sensor is selective to the desired species for detection. The existing EMPAS device requires an advance in design engineering to render it more user-friendly. Nevertheless, this work has clearly demonstrated that a more robust sensor using MEG-Cl and a bacteria-sensitive aptamer could be successfully developed for practical employment in the dairy industry for rapid evaluation of milk samples.

## Figures and Tables

**Table 1:** Frequency shifts for EMPAS crystals exposed to various concentrations of *E. coli* in either PBS or milk.

Crystal Coating	*E. coli* (CFU/mL)	Δ*f* in PBS (kHz)	Δ*f* in Milk (kHz)
Bare	1 × 10^8^	17 ± 4	27 ± 2
MEG-Cl	1 × 10^8^	0.5 ± 0.8	N/A
MEG-Aptamer	0	N/A	1.3 ± 0.3
	1 × 10^2^	1.2 ± 0.2	3.0 ± 0.7
	1 × 10^3^	2.4 ± 0.4	5.0 ± 0.2
	1 × 10^5^	4.8 ± 0.1	7.6 ± 0.3
	1 × 10^7^	8.4 ± 0.6	10.7 ± 0.2

**Table 2:** An overview of aptasensors used for the detection of *E. coli*.

*E. coli* Strain	Material Platform	Method of Detection	LOD, CFU/mL	Linear Range, CFU/mL	Food Sample/Recovery %	Reference
*Acoustic biosensors*						
8739 (Crooks strain)	Gold-thiolated aptamer	QCM	10^7^	NA	NA	[18]
O157:H7	Gold-MHDA-streptavidin, biotinylated aptamer	QCM	1.46 × 10^3^	5 × 10^2^–5 × 10^5^	NA	[13]
KCTC 2571	Gold-biotinylated thiol-streptavidin-biotinylated aptamer	QCM	10^4^	10^5^–10^8^	NA	[19]
DH5α	SiO_2_-MEG-NH_2_-Aptamer	EMPAS	8	10–10^7^	Not diluted milk/127.4	This work
*Electrochemical biosensors*						
O78:K80:H11	SCPE/MWCNTs/TPA	EIS	10	10–10^6^	Water, guava, litchi, mango juices, milk/NA	[36]
O157:H7	SCPE/BC –Ni	EIS	10	1–10^5^	Tap water, vegetable, carrot, orange juices, stool/NA	[37]
O157:H7	3D-IDEA	EIS	2.9 × 10^2^	10–10^5^	Water/92.3	[38]
BL21	IDE	EIS	9	25–10^3^	NA	[15]
NA	Au@MoS_2_–PANI nanocomposite	EIS, DPV	10	10–10^7^	Urine/90–110	[39]
CECT 675	SWCNTs-RNA aptamers	Potentiometry	6–26	4–2.4 × 10^4^	Filtrated milk, apple juice/NA	[40]
O157:H7	Gold-Aptamer/MCH/MB@MI nanocomposite	DPV	32	10^2^–10^7^	Milk/88.1–115.3	[41]
*Optical biosensors*						
O157:H7	CdSe/ZnS-QD	Fluorescence	10^2^	10^2^–10^7^	NA	[11]
O157:H7	PDA vesicles	Colorimetry	10^4^	10^4^–10^8^	Fecal samples in PBS/NA	[42]
O157:H7	AuNPs	Colorimetry	10^5^	10^6^–10^7^	NA	[43]
ATCC 25922	AuNPs	Colorimetry	10^5^	10^5^–10^8^	Meat/NA	[44]
O157:H7	Cu-MOF NPs/sandwich assay with two aptamers	Colorimetry	2	16–1.6 × 10^6^	Milk/filtrated/20x diluted with water/96–102.6	[45]
MTCC 1698	GO-AuNPs	Colorimetry	10	10–10^4^	Coconut water, litchi juice (both 10x diluted), bread homogenized and filtrated/NA	[46]
O157:H7	AuNPs	Colorimetry	147	1.2 × 10^2^–9 × 10^3^	Tap water/90.2–96.7	[47]

Abbreviations: 3D-IDEA: three-dimensional interdigitated electrode array; AuNPs: gold nanoparticles; BC–Ni: boron-carbon nanorods decorated by nickel nanoparticles; Cu-MOF NPs: copper-based metal-organic framework nanoparticles; DPV: differential pulse voltammetry; EMPAS: electromagnetic piezoelectric acoustic sensor; EIS: electrochemical impedance spectroscopy; GO-AuNPs: graphene oxide coated AuNPs; IDE: interdigitated electrode; MB: methylene blue; MCH: 6-mercaptohexanol; MI: magainin I; MHDA: 16-mercaptohexadecanoic acid; MWCNTs: multi-walled carbon nanotubes; PANI: polyaniline; PBS: phosphate buffer saline; PDA: polydiacetylene; QCM: quartz crystal microbalance; QD: quantum dot; SCPE: screen printed electrode; SWCNTs: single-walled carbon nanotubes; TPA: terephthalaldehyde.

## Data Availability

Not applicable.
